# Supplementary material for: An unsupervised explainable AI reveals oligonucleotide-usage zones matching 2,000 chromosome bands
Source: Life Sci Alliance. 2026 Nov 5;10(1):e202603782. doi: 10.26508/lsa.202603782 (PMC13740143; doi:10.26508/lsa.202603782)
Supplement: Supplementary file 1 [file LSA-2026-03782_Supplemental_Data_1.pdf]

**Supplementary data　Genomic coordinates of centromeric and pericentromeric constitutive heterochromatin regions.**

| | Start* | End* |
|---|---|---|
| chr1 | 120990001 | 144000000 |
| chr2 | 82990001 | 98000000 |
| chr3 | 90290001 | 96500000 |
| chr4 | 48990001 | 55300000 |
| chr5 | 44990001 | 51100000 |
| chr6 | 57990001 | 64100000 |
| chr7 | 56490001 | 66700000 |
| chr8 | 43790001 | 46900000 |
| chr9 | 44690001 | 77400000 |
| chr10 | 38490001 | 43200000 |
| chr11 | 48090001 | 55200000 |
| chr12 | 34190001 | 38100000 |
| chr13 | 1 | 18300000 |
| chr14 | 1 | 13100000 |
| chr15 | 1 | 21000000 |
| chr16 | 32590001 | 52300000 |
| chr17 | 21790001 | 28000000 |
| chr18 | 15490001 | 21200000 |
| chr19 | 24290001 | 30300000 |
| chr20 | 26290001 | 33000000 |
| chr21 | 1 | 11400000 |
| chr22 | 1 | 21000000 |
| chrX | 54890001 | 64000000 |
| chrY | 10490001 | 62100000 |

*Start and end positions on the genome sequences. These coordinates were used to remove centromeric and pericentromeric constitutive heterochromatin sequences in order to extract euchromatic sequences. As explained for Fig. 1, the BLSOM analysis was conducted by placing a 1-Mb window beginning at the start of the sequence and shifting it in 10-kb increments to calculate oligonucleotide compositions. Consequently, when the resulting BLSOM X-Y coordinates are plotted along the chromosomes and compared with the UCSC 850-band positions (Figs. 4, 6, and 7), it is important to note that the BLSOM analysis represents a moving-window analysis. The oligonucleotide composition of the first 1-Mb window corresponds to the composition at the +500-kb position. Accordingly, the genomic positions shown in these three distribution plots and the related Supplementary Figs. have been corrected to account for this positional offset.
