# Supplementary material for: An unsupervised explainable AI reveals oligonucleotide-usage zones matching 2,000 chromosome bands
Source: Life Sci Alliance. 2026 Nov 5;10(1):e202603782. doi: 10.26508/lsa.202603782 (PMC13740143; doi:10.26508/lsa.202603782)
Supplement: Supplementary file 2 [file LSA-2026-03782_review_history.pdf]

An unsupervised explainable AI reveals oligonucleotide-usage zones matching 2,000 chromosome bands

Toshimichi Ikemura, Yuki Iwasaki, Kennosuke Wada, Yoshiko Wada, and Takashi Abe
DOI: https://doi.org/10.26508/lsa.202603782

Corresponding author(s): Toshimichi Ikemura, Nagahama Institute of Bio-Science and Technology

Review Timeline:

Submission Date:	2026-05-24
Editorial Decision:	2026-08-12
Revision Received:	2026-09-03
Editorial Decision:	2026-10-06
Revision Received:	2026-10-14
Accepted:	2026-10-14

Scientific Editor: Sarita Hebbar

Transaction Report:

(Note: With the exception of the correction of typographical or spelling errors that could be a source of ambiguity, letters and reports are not edited. The original formatting of letters and referee reports may not be reflected in this compilation.)

August 12, 2026

Re: Life Science Alliance manuscript #LSA-2026-03782-T

Prof. Toshimichi Ikemura
Nagahama Institute of Bio-Science and Technology
Bioscience
Tamura-cho 1266
１６９－１６
Nagahama, Shiga 526-0829 526-0829
Japan

Dear Dr. Ikemura,

Thank you for submitting your manuscript entitled "Unsupervised AI reveals similar oligonucleotide-usage zones matching the prophase chromosome bands" to Life Science Alliance. We apologise for the delay in communicating a decision, due to reviewer availability issues.

Your manuscript was evaluated by two experts whose comments are appended below. As you will note, both reviewers find your work interesting but have raised significant conceptual and technical concerns. Overall we invite you to submit a revised ms addressing the reviewers' concerns.

We agree that you must completely address all the concerns of Reviewer 1 regarding technical clarifications (points 3-5), and must explicitly state the rationale for focus on Chr21 and 22.
We concur with Reviewer 1 (points 1, 2) that you must address their points on the sensitivity of the sliding window and size of the neural network as suggested.
Reviewer 2 raises a valid concern on the functional significance of the AI-identified zones. We suggest that you acknowledge this concern and expand on it in the discussion.

When submitting the revision, please include a letter addressing the reviewers' comments point by point. While a rebuttal must respond to all points in some form, additional experiments to resolve these points, other than indicated above, will not be required.

I would be happy to discuss the revision in more detail via email or phone/videoconferencing. Please let me know which option you prefer, if any.

To upload the revised version of your manuscript, please log in to your account: https://lsa.msubmit.net/cgi-bin/main.plex

You will be guided to complete the submission of your revised manuscript and to fill in all necessary information. Please get in touch in case you do not know or remember your login name.

The typical timeframe for revisions is three months. Please note that papers are generally considered through only one revision cycle, so strong support from the referees on the revised version is needed for acceptance.

While you are revising your manuscript, please also attend to the below editorial points to help expedite the publication of your manuscript. Please direct any editorial questions to the journal office.

Thank you for this interesting contribution to Life Science Alliance. We hope that the comments below will prove constructive as your work progresses, and we are looking forward to receiving your revised manuscript.

Sincerely,

Sarita Hebbar, PhD
Scientific Editor
Life Science Alliance
http://www.lsajournal.org

A. THESE ITEMS ARE REQUIRED FOR REVISIONS

-- A letter addressing the reviewers' comments point by point.

-- An editable version of the final text (.DOC or .DOCX) is needed for copyediting (no PDFs).

-- High-resolution figure, supplementary figure and video files uploaded as individual files: See our detailed guidelines for preparing your production-ready images, https://www.life-science-alliance.org/authors

-- Summary blurb (enter in submission system): A short text summarizing in a single sentence the study (max. 200 characters including spaces). This text is used in conjunction with the titles of papers, hence should be informative and complementary to the title and running title. It should describe the context and significance of the findings for a general readership; it should be written in the present tense and refer to the work in the third person. Author names should not be mentioned.

-- By submitting a revision, you attest that you are aware of our payment policies found here: https://www.life-science-alliance.org/copyright-license-fee

B. MANUSCRIPT ORGANIZATION AND FORMATTING:

Full guidelines are available on our Instructions for Authors page, https://www.life-science-alliance.org/authors

We encourage our authors to provide original source data, particularly uncropped/-processed electrophoretic blots and spreadsheets for the main figures of the manuscript. If you would like to add source data, we would welcome one PDF/Excel-file per figure for this information. These files will be linked as supplementary "Source Data" files.

IMPORTANT: It is Life Science Alliance policy that if requested, original data images must be made available. Failure to provide original images upon request will result in unavoidable delays in publication. Please ensure that you have access to all original microscopy and blot data images before submitting your revision.

Reviewer #1 (Comments to the Authors (Required)):

In this work, Ikemura et al. employ Batch-Learning Self-Organizing Map (BLSOM), an unsupervised and explainable AI method, to analyze oligonucleotide odds ratios across approximately 280,000 overlapping 1-Mb fragments of the human T2T genome. The authors demonstrate that four distinct oligonucleotide feature sets partition euchromatin into approximately 2,000 distinct zones with similar sequence usage. These AI-identified zones are then shown to correspond to roughly 2,000 Giemsa bands observable at the highest-resolution human chromosome banding pattern at prophase. The paper is well-written and interesting to read, and I am glad to see less common AI techniques applied to biology. My comments for this work are as follows:

1. Most of the experiments were performed with 1MB sliding window, which was shifted by 10kb. Was there a particular reason for choosing this? How sensitive is the zone count to the choice of window size, i.e., would you be able to identify 2,000 zones if you chose 500k or 2MB sliding window?

2. The authors mention that the size of the model is chosen so that each node contains 10 sequences. How much do the results depend on the size of the neural network? Showing the performance of a model with different sizes on one of the experiments would be interesting.

3. The weights of the model were initialized with PCA, but it is not actually quantified how much this helps with the model performance. I would expect an ablation study of this to show that a randomly initialized model performs suboptimally. The authors say that initializing the weights via PCA helps the model be deterministic, but the same should be achievable by specifying a random seed and then initializing the weights randomly. Or if using a PCA is a standard way of initializing weights in SOMs, could you provide some references for that?

4. For how many steps was the network trained? How do you determine that the training has converged? Did you track quantization error or topographic error, and if so, could you provide them as supplemental figures? This would clearly show that the model was not undertrained or overtrained.

5. Better description of the training process. From my understanding, training SOMs is significantly more computationally efficient than training models like LLMs, but it would be good to see how much time and memory were needed, and what hardware was used.

6. Most of the analysis was done on chromosomes 21 and 22. Was there a particular reason for choosing them, aside from them being the shortest?

7. Figure 5Biii (PentaOdds) - it seems like there is something wrong with the caption of that subfigure, like it is incorrectly

rendered.

Reviewer #2 (Comments to the Authors (Required)):

Using an unsupervised and explainable AI (BLSOM), the authors uncovered regions of exceptionally high penta- and hexanucleotide frequencies in the human genome. They computed the odds ratio (observed/expected) profiles of all 1-Mb euchromatic fragments in the human genome and found nearly 2,000 distinct zones, which is highly unexpected because the expected numbers for penta- and hexanucleotides are very different (1,024 vs 4,096 variables). Under the assumption that these ~2,000 zones represent biologically meaningful segmentations, comparable structures would be expected to emerge when other oligonucleotide types are analyzed. Consistent with this expectation, CG-containing penta- and hexanucleotides (244 and 1,185 variables) produced comparable ~2,000 zones. They therefore argued that the observed segmental structures reflect fundamental functional divisions within the human genome. Human chromosomes exhibit well-established Giemsa-banding patterns comprising 850 bands at prometaphase and 2,000 bands at prophase. From the single-nucleotide coordinates for the 850 bands, the authors identified a diagnostic oligonucleotide set that distinguishes Giemsa-negative and -positive regions. Computational pseudo-band reconstruction based on this set generated genome segmentations that more closely paralleled the AI-derived ~2,000 clusters than the 850 bands. They therefore concluded that their study captured characteristic features of chromosomal bands.
The study contains some interesting discoveries. However, except for the Giemsa-banding pattern data, the authors could not find any functional data to support the functional significance of their findings. Indeed, they could not say what these ~2000 zones actually do. Also, although the title of the paper says "unsupervised and explainable AI", the study used only the BLSOM software package, but no other AI tools.

Response to Reviewers

Reviewer 1

Comment:

I am glad to see less common AI techniques applied to biology.

Response:

The Editor noted that it was difficult to find suitable reviewers for this manuscript. I would first like to express my sincere gratitude to the reviewer for taking the time to evaluate this interdisciplinary study. Thank you also for your comment, “I am glad to see less common AI techniques applied to biology,” from the perspective of an AI expert. As you noted, our study applies AI techniques that are currently less common in mainstream AI research to biological questions, and we consider this to be one of the distinctive features of the study. I believe that diversity and originality in research are important for scientific progress. In my own experience, several of my papers that have received more than 1,000 citations addressed research questions for which there were few comparable studies at the time of publication (see my Google Scholar profile under “Toshimichi Ikemura”). This experience has encouraged me to continue pursuing distinctive research directions, even when they appear to lie outside the current mainstream. I hope you will understand that this perspective underlies the present study. Please forgive me if the following response is primarily from a biologist’s perspective.

Comment 1:

Most of the experiments were performed with 1MB sliding window, which was shifted by 10kb. Was there a particular reason for choosing this? How sensitive is the zone count to the choice of window size, i.e., would you be able to identify 2,000 zones if you chose 500k or 2MB sliding window?

Response:

We took a data-driven approach, first obtaining the AI results and then exploring their biological meaning, rather than starting with a specific model or hypothesis and testing it. Because of this approach, we did not initially explain clearly why we chose a 1-Mb window. However, the reviewer’s comment made us realize that stating from the outset that the 1-Mb window was chosen for chromosome-band analysis would make the study easier for readers to understand. We have therefore added this explanation to the revised manuscript (Lines 57–59). We thank the reviewer for this valuable comment.

Following the reviewer's comment, we performed an additional analysis using a 2-Mb window and again identified approximately 2,000 zones. In addition, we obtained the useful finding that the black-and-white boundaries in the U-matrix became slightly clearer.

The reason for using a 10-kb moving step is explained in the original manuscript (Lines 149–157; revised manuscript, Lines 139–147). Our preliminary analysis showed that a step size of 100 kb substantially reduced computation time but made the U-matrix pattern less clear. A moderate increase, for example to 20 kb, might reduce computation time while maintaining the clarity of the U-matrix pattern, but we did not perform such detailed analyses to optimize computation time. Rather, we consistently used a 10-kb step size for all analyses because this criterion is simple and easy for readers to understand.

Comment 2:

The authors mention that the size of the model is chosen so that each node contains 10 sequences. How much do the results depend on the size of the neural network? Showing the performance of a model with different sizes on one of the experiments would be interesting.

Response:

Our preliminary analysis showed that using 100 sequences per node greatly shortened computation time but made the U-matrix patterns less clear. This was why we chose 10 sequences per node despite the longer computation time. Using 20 sequences per node might shorten computation time without greatly reducing the resolution, but this choice would require additional justification. Rather than optimizing computation time, we consistently used 10 sequences per node for all analyses because this criterion is simple and easy for readers to understand.

Comment 3:

The weights of the model were initialized with PCA, but it is not actually quantified how much this helps with the model performance. I would expect an ablation study of this to show that a randomly initialized model performs suboptimally. The authors say that initializing the weights via PCA helps the model be deterministic, but the same should be achievable by specifying a random seed and then initializing the weights randomly. Or if using a PCA is a standard way of initializing weights in SOMs, could you provide some references for that?

Response:

PCA-based initialization is an established approach for SOMs and is described in Kohonen's standard reference (Self-Organizing Maps, 3rd ed., 2001). Kohonen noted that PCA initialization can

substantially accelerate SOM training, which was one reason for our choice. In addition, PCA provides a reproducible, data-driven initial configuration. Although proper clustering can also be achieved using a random seed, we preferred PCA because it avoids an arbitrarily chosen random configuration.

For very large datasets, however, PCA itself is computationally demanding. For example, PCA of more than 260,000 sequences with 4,096 hexanucleotide variables requires an extremely long computation time. In preliminary analyses of pentanucleotides, we had reduced the number of sequences used for PCA initialization to 1/10 or even 1/100 and observed no appreciable change in the final SOM clustering. This was not clearly described in the original manuscript. We have now added this information, including the actual reduction level for the respective BLSOMs, to the Materials and Methods (Lines544–550).

Comment 4:
For how many steps was the network trained? How do you determine that the training has converged? Did you track quantization error or topographic error, and if so, could you provide them as supplemental figures? This would clearly show that the model was not undertrained or overtrained.

Response:
The number of training steps varied depending on the oligonucleotide type and was typically 300–600 for the major BLSOM analyses in this study. Each training step ended when the convergence criterion was met or the predefined maximum number of iterations was reached, after which the Neighbor Size was updated. The number of iterations within each step was not recorded in the calculation logs. Convergence was evaluated using a predefined threshold, primarily a Square of Weight Delta value of 0.00001.

We did not track quantization error or topographic error. Such rigorous evaluation methodologies were extensively developed by experts in mathematics, statistics, and computer science during the establishment and maturation of AI methodologies around the period of the second AI boom. These efforts contributed substantially to establishing SOM and related approaches as textbook-level methodologies. Our group has used these established AI methodologies as tools for addressing biological questions from a biologist's perspective. Using this approach, we have published approximately 30 peer-reviewed papers in a variety of journals, including *Genome Research, The ISME Journal, Life Science Alliance, Gene, and DNA Research.* In these studies, we have not been asked to conduct such highly specialized verification. The present study is not intended as a

methodological paper proposing a new AI algorithm.

Furthermore, from a biologist's perspective, highly specialized statistical measures of AI performance alone would not necessarily provide convincing evidence for the biological reliability of the AI findings, either to the biologists conducting the study or to most biological readers. We therefore considered it more important to validate the major conclusions using approaches that biologists can readily understand. It would be even more desirable if the conclusions were supported by methods other than AI. We adopted this approach, and Reviewer 2 recognized that it was successfully implemented in this study. We therefore quote Reviewer 2's evaluation below in parentheses and italics.

[They computed the odds ratio (observed/expected) profiles of all 1-Mb euchromatic fragments in the human genome and found nearly 2,000 distinct zones, which is highly unexpected because the expected numbers for penta- and hexanucleotides are very different (1,024 vs 4,096 variables). Under the assumption that these ~2,000 zones represent biologically meaningful segmentations, comparable structures would be expected to emerge when other oligonucleotide types are analyzed. Consistent with this expectation, CG-containing penta- and hexanucleotides (244 and 1,185 variables) produced comparable ~2,000 zones. They therefore argued that the observed segmental structures reflect fundamental functional divisions within the human genome. Human chromosomes exhibit well-established Giemsa-banding patterns comprising 850 bands at prometaphase and 2,000 bands at prophase. From the single-nucleotide coordinates for the 850 bands, the authors identified a diagnostic oligonucleotide set that distinguishes Giemsa-negative and -positive regions. Computational pseudo-band reconstruction based on this set generated genome segmentations that more closely paralleled the AI-derived ~2,000 clusters than the 850 bands. They therefore concluded that their study captured characteristic features of chromosomal bands.]

When considering why Reviewer 1 regarded this study as an AI methodology paper, we realized that our message to AI experts appeared only at the end of the Discussion (Original manuscript Lines 526–550). We have therefore added an explanation in the Introduction of the role of biologists in AI-based research (Lines 104–110), together with two additional references (Lines 594–596 and 642–645). We believe that this revision will help AI experts better understand the basic issues in this study and the significance of our approach.

Comment 5:

Better description of the training process. From my understanding, training SOMs is significantly more computationally efficient than training models like LLMs, but it would be

good to see how much time and memory were needed, and what hardware was used.

Response:
The BLSOM calculations were performed mainly on a Dell Precision 7920 Tower equipped with 40 CPU cores and 64 GB of memory. We did not routinely use all 40 cores at full capacity, and computation time varied among analyses but was typically 1–5 days. All analyses used less than half of the available 64 GB of memory, and the actual memory usage of individual calculations was not recorded. We have added part of this information to the Materials and Methods (Lines 552–555).

Comment 6: *Most of the analysis was done on chromosomes 21 and 22. Was there a particular reason for choosing them, aside from them being the shortest?*

Response:
The BLSOM analyses themselves were performed using sequences from all chromosomes (original manuscript, Lines 109–110; revised manuscript, Lines 114–115). Chromosomes 21 and 22 were selected as representative examples to visualize the relationship between positions on the BLSOM and their corresponding positions along the genome. Because longer chromosomes would require much wider figures to display these relationships clearly, the two shortest chromosomes were chosen to make effective use of figure space.

In addition, chromosomes 21 and 22 provide biologically contrasting examples: chromosome 21 has a relatively low gene density, whereas chromosome 22 has a relatively high gene density. We have added this point concerning gene density to the revised manuscript (original manuscript, Lines 161–163; revised manuscript, Lines 151–153). The generality of the conclusions obtained from chromosomes 21 and 22 was also examined across the other chromosomes using heatmap analyses (original manuscript, Lines 164–169; revised manuscript, Lines 156–160).

Comment 7:
Figure 5Biii (PentaOdds) - it seems like there is something wrong with the caption of that subfigure, like it is incorrectly rendered.

Response:
Thank you for pointing this out. The font in the caption of Fig. 5Biii was incorrectly rendered. We have corrected it in the revised manuscript.

Finally, I sincerely appreciate once again your review of our manuscript, which addresses an

interdisciplinary research topic for which it can be particularly difficult to find appropriate reviewers. Although we have entered the era of AI, many researchers in the life sciences still hesitate to review studies using AI methods with which they have little direct experience, even when the biological issues under investigation are closely related to their expertise. I believe, however, that a new generation of researchers who can work in both experimental biology and AI will emerge in the near future. One of the aims of our research is to contribute to the development of such researchers.

Reviewer 2

Response:
We thank the reviewer for the positive evaluation of our efforts to validate the reliability of the AI analysis from a biologist's perspective, which was our primary concern in this study. We next address the reviewer's comment on the limitations of our study.

Comment 1:
However, except for the Giemsa-banding pattern data, the authors could not find any functional data to support the functional significance of their findings. Indeed, they could not say what these ~2000 zones actually do. Also, although the title of the paper says "unsupervised and explainable AI", the study used only the BLSOM software package, but no other AI tools.

Response:
It is difficult for a computational study alone to provide new functional data. Chromosome bands and their functions have long been a subject of my experimental and computational research, and understanding chromosome-band structure as functional segmentation remains an important biological issue also in the respective research field.

Although chromosome bands are widely known as cytogenetic landmarks, increasing evidence indicates that they also represent functionally distinct genomic regions. As described in the original manuscript, different band types differ in gene expression, DNA replication timing, and chromatin organization associated with transcriptional regulation (original manuscript, Lines 89–93; revised manuscript, Lines 87–90). In the present study, we quantitatively characterized sequence features of chromosome bands as diagnostic oligonucleotides, providing a numerical connection between oligonucleotide sequences and chromosome-band structure. Representative examples include TA-containing oligonucleotides, which are associated with chromatin condensation and transcriptional repression (original manuscript, Lines 363–369; revised manuscript, Lines 334–338), and CG-

containing oligonucleotides, which have well-established epigenomic functions through cytosine methylation (original manuscript, Lines 369–372; revised manuscript, Lines 338–341). Thus, the diagnostic oligonucleotides identified here provide a sequence-level link between chromosome-band structure and known genomic functions.

However, the above explanation is clearly not sufficient to claim that we discovered a highly novel function of chromosome bands. However, as a group that has long aimed to understand chromosome-band functions, we believe that this study has unexpectedly provided a new approach toward this goal. We think that developing a method for discovering new knowledge is also important, as is the discovery of new knowledge itself. The reviewer's critical comment regarding function made us realize that the original manuscript did not sufficiently explain the significance of this study for understanding chromosome-band functions. We have therefore revised this part, with our future studies in mind. The following explanatory text in parentheses was added to the Discussion under the heading "Bridging classical cytogenetics and modern AI-based genomics" (Lines 477–493).

[*Bridging classical cytogenetics and modern AI-based genomics: Chromosome-band research has a much longer history than genome sequencing, and extensive banding patterns have been reported for many higher eukaryotes. However, systematic analysis of their sequence characteristics has been difficult because individual band boundaries were not defined at nucleotide-level resolution. In the human genome, numerous mapped BAC clones enabled chromosome-band coordinates to be defined at the sequence level, making the present analysis possible. The human genome is exceptionally well characterized, with extensive experimental and functional genomic data available. Importantly, our AI analysis shows that chromosome-band-like structures can be obtained from genome sequence alone. This suggests that similar AI-based approaches could relate chromosome-band structures to nucleotide-level sequence features in a wide range of higher eukaryotes.*
For species evolutionarily distant from humans, the diagnostic oligonucleotides available by the AI-approach are likely to differ, and identifying such species-specific features could help investigate chromosome-band functions at the oligonucleotide-sequence level. We therefore believe that our approach provides a framework for integrating microscopic chromosome studies with nucleotide-level genome analyses and for investigating Mb-level functional genome organization across diverse species.]

According to the reviewer's comment and the LSA guidelines, we revised the title of the manuscript (Lines 1–2).

October 6, 2026

RE: Life Science Alliance Manuscript #LSA-2026-03782-TR

Prof. Toshimichi Ikemura
Nagahama Institute of Bio-Science and Technology
Bioscience
Tamura-cho 1266
１６９－１６
Nagahama, Shiga 526-0829 526-0829
Japan

Dear Dr. Ikemura,

Thank you for submitting your revised manuscript entitled "An unsupervised explainable AI reveals oligonucleotide-usage zones matching 2,000 chromosome bands".

Your manuscript was evaluated by Reviewer 1 whose comments are appended below. As you will read, this reviewer finds an improvement in your revised version and notes that their concerns have been addressed either by the revisions or clarifications provided in the rebuttal letter.

Based on their overall assessment, we would be happy to publish your paper in Life Science Alliance pending final revisions necessary to meet our formatting guidelines.

MANUSCRIPT ORGANIZATION AND FORMATTING:

To avoid unnecessary delays in the acceptance and publication of your paper, please read the following information carefully. Full guidelines are available on our Instructions for Authors page, https://www.life-science-alliance.org/authors

Submission of a paper that does not conform to Life Science Alliance guidelines will delay the acceptance of your manuscript.

-Please include a legend for Figure S6.
-Please add callouts for Figures 1C; S1A-C; S2A-B and S4B to your main manuscript text.
-Your manuscript refers to details in supplementary methods (lines 544, 843). Kindly incorporate these details to the section titled 'Materials and Methods' in the main manuscript.
-Images in Figure 3Di and Diii appear identical to images in Figures S3 Bi and Biii. Please provide appropriate clarification with original images as needed, or please correct your figure legend and state the re-utilisation of images in both figure legends.
-Thank you for providing a statement on data availability. Please note that all codes and source data to reproduce this work should be listed in a structured manner in the "Data Availability" section. Do refer to our guidelines: https://www.life-science-alliance.org/editorial-policies#data-sharing
-Please add a Summary Blurb/Alternate Abstract in our system.
-Please add the X and Bluesky handles of your host institute/organization, as well as your own, and/or one of the authors, in our system.
-Please be sure that the authorship listing and order is correct.

We encourage our authors to provide original source data, particularly uncropped/-processed electrophoretic blots and spreadsheets for the main figures of the manuscript. If you would like to add source data, we would welcome one PDF/Excel-file per figure for this information. These files will be linked as supplementary "Source Data" files.

It is Life Science Alliance policy that if requested, original data images must be made available to the editors. Failure to provide original images upon request will result in unavoidable delays in publication. Please ensure that you have access to all original data images prior to final submission.

If you are planning a press release on your work, please inform us immediately to allow informing our production team and scheduling a release date.

We welcome submissions of potential cover images for the issue of LSA in which your work would appear. If you have high quality images associated with this work, please feel free to email these, with a caption, to the journal office.

LSA encourages authors to provide a 30-60 second video where the study is briefly explained. These videos will be appear

embedded with the manuscript online at Life Science Alliance, and on social media to promote the published paper and authors (for examples, see https://docs.google.com/document/d/1-UWCfbE4pGcDdcgzcmiuJl2XMBJnxKYeqRvLLrLSo8s/edit?usp=sharing). Corresponding or first-authors are welcome to submit the video. Please submit only one video per manuscript. The video can be emailed to contact@life-science-alliance.org

FINAL FILES:

To upload the final version of your manuscript, please log in to your account: https://lsa.msubmit.net/cgi-bin/main.plex
You will be guided to complete the submission of your revised manuscript and to fill in all necessary information. Please get in touch in case you do not know or remember your login name.

The following items are required for acceptance.

-- An editable version of the final text (.DOC or .DOCX) is needed for copyediting (no PDFs).

-- High-resolution figure, supplementary figure and video files uploaded as individual files: See our detailed guidelines for preparing your production-ready images, https://www.life-science-alliance.org/authors

-- Summary blurb (enter in submission system): A short text summarizing in a single sentence the study (max. 200 characters including spaces). This text is used in conjunction with the titles of papers, hence should be informative and complementary to the title. It should describe the context and significance of the findings for a general readership; it should be written in the present tense and refer to the work in the third person. Author names should not be mentioned.

The license to publish form must be signed before your manuscript can be sent to production. A link to the license to publish form will be available to the corresponding author only. Please take a moment to check your funder requirements.

Reviews, decision letters, and point-by-point responses associated with peer-review at Life Science Alliance will be published, alongside the manuscript. If you do want to opt out of having the reviewer reports and your point-by-point responses displayed, please let us know immediately.

Thank you for your attention to these final processing requirements. Please revise and format the manuscript and upload materials as soon as you are able.

Thank you for this interesting contribution to the literature. We look forward to publishing your paper in Life Science Alliance.

Sincerely,

Sarita Hebbar, PhD
Scientific Editor
Life Science Alliance
http://www.lsajournal.org

Reviewer #1 (Comments to the Authors (Required)):

I thank the authors for their work. All of my comments have been addressed. Thanks to the detailed explanations provided by the authors, I see that some of my comments were based on my insufficient understanding of the common practices in training SOMs (e.g., Comments 3 and 4). Overall, I find the manuscript greatly improved.

October 14, 2026

RE: Life Science Alliance Manuscript #LSA-2026-03782-TRR

Prof. Toshimichi Ikemura
Nagahama Institute of Bio-Science and Technology
Bioscience
Tamura-cho 1266
１６９－１６
Nagahama, Shiga 526-0829 526-0829
Japan

Dear Dr. Ikemura,

Thank you for submitting your Research Article entitled "An unsupervised explainable AI reveals oligonucleotide-usage zones matching 2,000 chromosome bands", and we acknowledge that you have addressed our formatting requests. We have noted that you incorporated relevant citations to the concise description, from the supplementary (methods) file, to the main text.

It is a pleasure to let you know that your manuscript is now accepted for publication in Life Science Alliance. Congratulations on this interesting work.

The final published version of your manuscript will be deposited by us to PubMed Central upon publication.

Your manuscript will now progress through copyediting and proofing. It is journal policy that authors provide original data upon request.

Your article will publish open access upon publication under a CC-BY license.

Reviews, decision letters, and point-by-point responses associated with peer-review at Life Science Alliance will be published, alongside the manuscript. If you do want to opt out of having the reviewer reports and your point-by-point responses displayed, please let us know immediately.

IMPORTANT: If you will be unreachable at any time, please provide us with the email address of an alternate author. Failure to respond to routine queries may lead to unavoidable delays in publication.

Scheduling details will be available from our production department. You will receive proofs shortly before the publication date. Only essential corrections can be made at the proof stage so if there are any minor final changes you wish to make to the manuscript, please let the journal office know now.

DISTRIBUTION OF MATERIALS:
Authors are required to distribute freely any materials used in experiments published in Life Science Alliance. Authors are encouraged to deposit materials used in their studies to the appropriate repositories for distribution to researchers.

You can contact the journal office with any questions, contact@life-science-alliance.org

Again, congratulations on a very nice paper. I hope you found the review process to be constructive and are pleased with how the manuscript was handled editorially. We look forward to future exciting submissions from your lab.

Sincerely,

Sarita Hebbar, PhD
Scientific Editor
Life Science Alliance
http://www.lsajournal.org
